# Altered heart-brain coupling in awake patients with isolated REM sleep behaviour disorder

**DOI:** 10.1038/s41531-026-01485-7

**Published:** 2026-07-23

**Authors:** Fosco Bernasconi, Julia van der Meer, Zehra Merchant, Jevita Potheegadoo, Marzia De Lucia, Claudio Bassetti, Carolin Schaefer, Olaf Blanke

**Affiliations:** 1 Laboratory of Cognitive Neuroscience, Neuro-X Institute, School of Life Sciences, Ecole Polytechnique Fédérale de Lausanne (EPFL), Geneva, Switzerland; 2 Sleep-Wake Epilepsy Center, Department of Neurology, Inselspital, University Hospital of Bern, University of Bern, Bern, Switzerland; 3 Brain-Body and Consciousness Laboratory, Department of Clinical Neuroscience, Lausanne University Hospital, University of Lausanne, Lausanne, Switzerland; 4 Center for Biomedical Imaging, Lausanne, Switzerland; 5 Clinic for Sleep Medicine, KSM Luzern, Lucerne, Switzerland; 6 Department of Neurology, Geneva University Hospitals, Geneva, Switzerland

**Keywords:** Biomarkers, Neurology, Neuroscience

## Abstract

Isolated REM sleep behaviour disorder (iRBD) represents a prodromal stage of alpha-synucleinopathies, frequently preceding Parkinson’s disease and dementia with Lewy bodies. Beyond the characteristic sleep disturbances, alterations in brain-body coupling, reflecting neural peripheral physiological interactions, have yet to be determined. Using whole-night polysomnography, we quantified brain-body coupling via heartbeat evoked potentials (HEPs) across wakefulness, NREM, and REM sleep. Individuals with iRBD (*n* = 13) were compared with healthy controls (*n* = 23), alongside analyses of ECG-derived features. During wakefulness, iRBD patients showed altered HEPs over frontal regions between 305 and 445 ms after the R-peak. No significant group differences were observed during NREM or REM sleep, and HEP amplitude alterations were not explained by ECG differences. These diurnal HEP changes may represent a novel quantitative biomarker for iRBD during wakefulness and, in the future, could serve as a marker for phenoconversion to alpha-synucleinopathy.

## Introduction

Rapid eye movement (REM) sleep behaviour disorder (RBD) is characterized by vocalizations, jerks, and complex motor behaviours during REM sleep, often associated with vivid, negatively toned, and aggressive dream content, accompanied by the loss of normal muscle atonia^1^. In the absence of other neurological symptoms, RBD is defined as isolated (iRBD) and recognized as one of the earliest clinical manifestations of α-synucleinopathies^2,3^. The prevalence of iRBD is documented to be approximately 1%, predominantly affecting older adults, particularly individuals over 60 years of age, and occurs more frequently in men than in women^4^. iRBD is a strong prodromal marker for Parkinson’s disease (PD), dementia with Lewy bodies (DLB), and rarely multiple system atrophy^1,5^. More than 80% of individuals with iRBD eventually develop one of these neurodegenerative disorders within a decade or more^5^. RBD is also a frequent feature of PD^6^ and a core symptom of DLB^7^. In these cases, it is categorized as secondary RBD. In PD, the presence of RBD has been associated with a more aggressive course of the disease, characterized by faster progression and more severe non-motor symptoms^8^.

Although iRBD is primarily associated with sleep disturbances, growing evidence also links autonomic dysfunction (i.e., dysautonomia) with iRBD^9^. Given that iRBD constitutes an early manifestation of α-synucleinopathies, the co-occurrence or early appearance of autonomic dysfunction likely reflects the underlying spread of pathology within central autonomic networks^9^. The spectrum of autonomic dysfunction in iRBD is broad. Clinical interviews revealed symptoms related to gastrointestinal, erectile, and urinary dysfunctions in iRBD^10–12^ and different cardiovascular abnormalities such as cardiac sympathetic denervation^13^, abnormal baroreflex sensitivity, and reduced heart rate variability (HRV) during REM sleep^14–17^. Findings on HRV in iRBD patients are heterogeneous, with some studies reporting alterations in low-frequency and/or high-frequency HRV spectra, indicative of cardiac sympathetic and parasympathetic dysfunction, respectively^18^. Other studies did not observe any abnormality in HRV^19,20^.

Growing evidence shows that the brain integrates different interoceptive (bodily) signals arising from the internal organs (for review see ref. ^21^. Hence, neural activity is modulated by cardiac^22–24^, gastric^25,26^, and breathing signals^27,28^. The ascending interoceptive signals, such as those from the heart, that give rise to a neural response are conveyed to the brain via cranial nerves (especially the vagal nerve) as well as spinal pathways^29^. These ascending interoceptive signals are processed in the brainstem and propagate via the thalamus to viscerosensory cortices such as the insula, somatosensory, and anterior cingulate cortex^22,29–32^. Even though the same regions and pathways, especially the brainstem and basal ganglia, are altered in iRBD patients^8,33–40^ it is currently unknown whether the neural interoceptive processing is altered in these individuals.

Several lines of evidence suggest that awake patients with PD are characterized by impairments in interoceptive processing, either in showing reduced awareness of their own heartbeats^41–43^ or an altered neural response to the heartbeat signal^44^. However, it has not been investigated whether these neural alterations in cardiac interoceptive processing are already present in iRBD (a prodromal stage of PD/DLB). Many recent studies investigating the brain processing of heartbeat signal in healthy individuals, analysed the so-called heartbeatevoked potentials (HEPs), derived from electroencephalographic (EEG) signals that are time-locked to the R-peak of the electrocardiogram (ECG). HEPs typically manifest as a negative deflection approximately 200–600 milliseconds after the R-peak and are most prominent over fronto-central regions^23,32,45–47^.

Leveraging whole-night polysomnography recordings conducted in thirty-six individuals, we conducted an exploratory study to investigate whether the brain processing of the heartbeat signal, indexed by HEPs, is altered in patients with iRBD vs. healthy control individuals (HC), and whether this differed between different periods of the sleep-wake cycle (NREM and REM sleep, and during wakefulness). Based on the prior evidence mentioned above, we hypothesized that the HEP amplitude would be altered in awake iRBD individuals, but not necessarily during sleep. In addition, we also expected a difference (independent of the group) in HEP amplitude when comparing wakefulness with sleep.

## Results

### Polysomnographic recordings

We analysed polysomnography recordings of patients with iRBD (iRBD: *N* = 13) and healthy controls (HC: *N* = 23). The demographic data did not show significant (X^2^ = 1.853; *p*-value = 0.173) differences in sex (iRBD: 2/11 (female/male); HC: 8/15 (female/male) between the two groups, but we observed a significant difference (*p*-value = 0.01) in age, with patients with iRBD being older (iRBD: 65.7 ± 7 years; HC: 57.3 ± 8.8 years).

### Heartbeat-evoked potentials (HEPs)

**Fig. 1:**
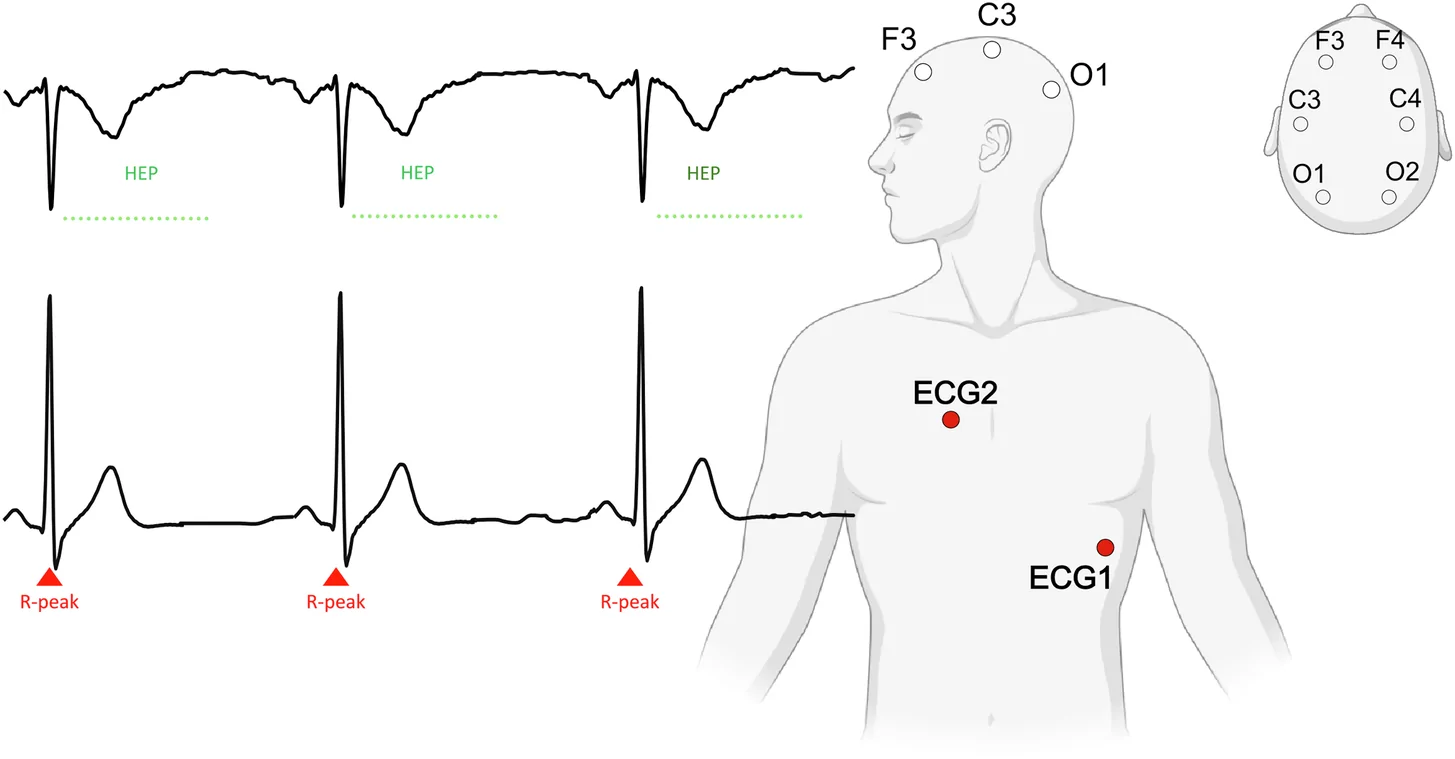
Polysomnography setup and HEPs. Illustration of the polysomnography setup, which includes the recording of the ECG (red dots) and six scalp EEG electrodes (green dots). HEPs are computed by time-locking the EEG signal to the R-peak of the ECG.

**Fig. 2:**
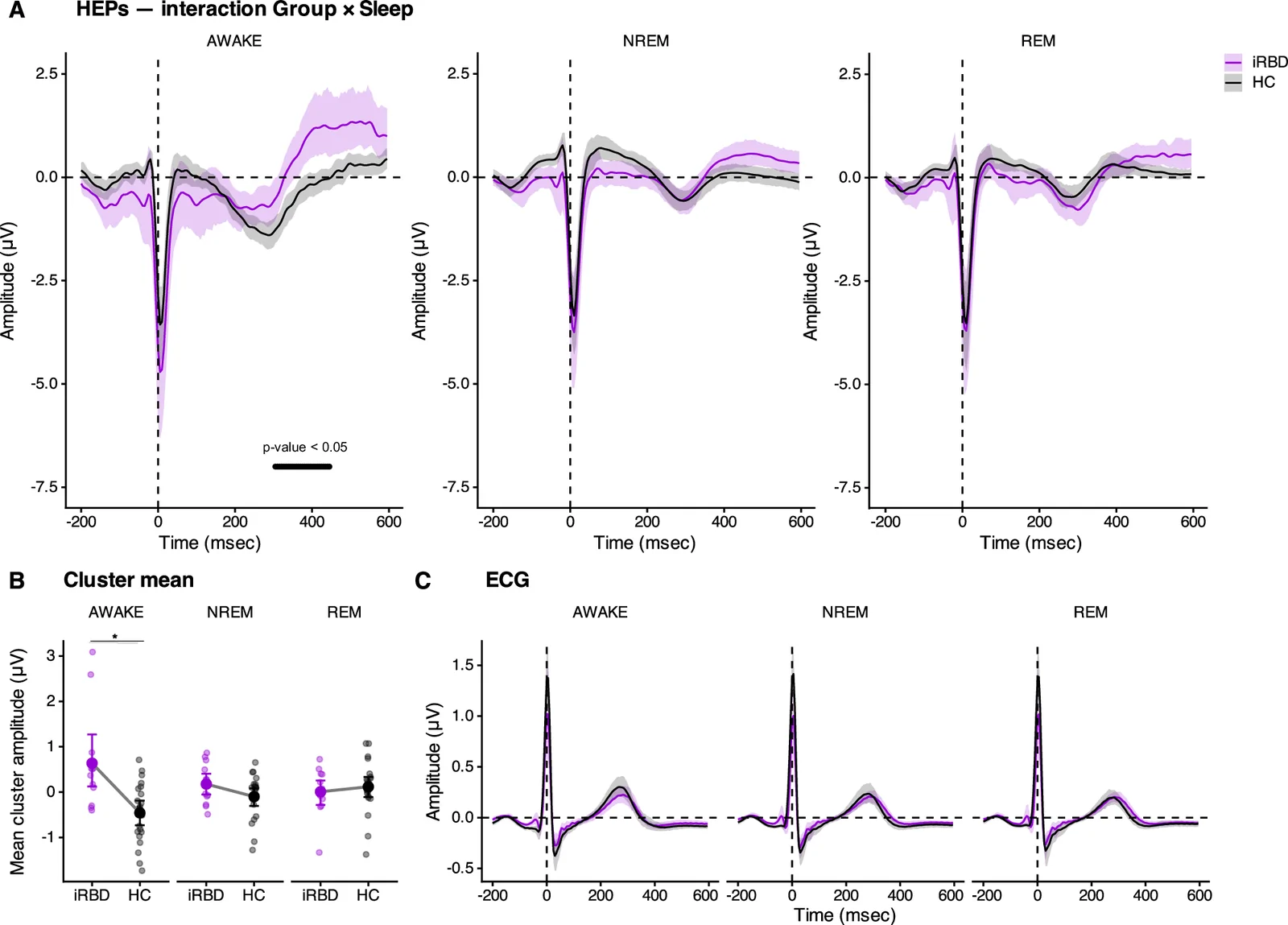
HEPs are altered in iRBD patients during wakefulness. **A** HEPs on electrode F3 (green circle on the EEG layout). During wakefulness (left panel) are altered in iRBD patients (purple) compared to controls (black), the black line represents the period of significant interaction (Group X wake-sleep stage; *p*-values < 0.05 and FDR-corrected) obtained through linear mixed models. The continuous lines represent the groups’ means, and the shaded areas represent the 95% CI. **B** Mean HEP amplitude over the period showing a significant interaction Group X wake-sleep stage. Post-hoc analyses showed a significant difference (*p*-value < 0.05; indicated by the asterisk) between groups during wakefulness, but not during sleep (NREM and REM). Each dot represents a subject; the larger dot represents the mean with the 95% CI. **C** ECG amplitude. We did not observe any significant interaction (Group X wake-sleep stage; *p*-values > 0.05; FDR-corrected).

To investigate alterations in the neural response to the heartbeat signal, we computed HEPs separately for each EEG channel (F3, F4, C3, C4, O1, O2) (Fig. 1), sleep-wake cycle (NREM, REM, wakefulness), in each individual’s data set. As expected, visual inspection of the HEPs in each sleep stage showed a predominant response over fronto-central electrodes, and the HEPs’ typical negative deflection (visible in the EEG signal) occurring approximately 200–600 milliseconds after the R-peak as defined based on the ECG recording (time-locking event) (Fig. 2A).

To assess whether HEP amplitude differs between iRBD patients and HC, and whether such amplitude difference varies across sleep-wake stages, we fitted linear mixed-effects models for each channel and time point independently. HEP amplitude served as the dependent variable, with Group (iRBD vs. HC) and sleep–wake stage (NREM, REM, wakefulness) included as fixed effects, along with their interaction term. A random intercept was specified for each individual to account for inter-individual variability, and a random slope was included to model variability across sleep stages. Age was included as a covariate of no interest.

### HEPs are altered in iRBD during wakefulness, but not during NREM and REM sleep

Our results showed a significant interaction (*p*-values < 0.05, FDR-corrected) between *Groups* (iRBD vs. HC) and *wake-sleep stages* (wakefulness, NREM, and REM), over the period 305 ms–445 ms after the R-peak (Fig. 2A). This interaction was specific to the frontal electrode, lateralized on the left side (electrode F3; also see Supplementary Materials). Post-hoc comparisons, computed on the mean of the whole significant interaction period (305 ms–445 ms), showed that during wakefulness, iRBD patients have altered HEP amplitudes compared to controls (Fig. 2A, B). This difference was not observed during NREM and REM sleep (Fig. 2A,B).

To exclude that these HEP differences might be related to ECG amplitude differences, we also investigated whether the ECG signal is modulated between the two subgroups and wake-sleep stages (same statistical model as for the EEG electrodes). This analysis showed no statistically significant interaction (*p*-value > 0.05; FDR-corrected) (Fig. 2C), suggesting that the altered HEPs observed in iRBD patients are not caused by a peripheral cardiac difference. Furthermore, periods of apnea and hypopnea were removed prior the HEP analyses and therefore cannot account for HEP amplitude differences (also see Supplementary Table 1).

### Duration of RBD symptoms associated with HEP amplitude alteration

We investigated whether the duration of the RBD symptoms is associated with the HEP amplitude alterations observed during wakefulness in iRBD patients. With this aim, we conducted an exploratory association between the duration of the RBD symptoms (from the onset of the symptoms till the day of the PSG; as dependent variable) and the HEP amplitude (mean of the 305 ms–445 ms significant cluster for electrode F3). The mean duration of the RBD symptoms was 2.474 years (SD ± 2.762). Our results show a marginally significant (F = 3.98, *p*-value = 0.07) positive association, with longer RBD symptom duration being associated with stronger HEP amplitude alterations. Future studies with a larger sample of patients are needed to corroborate these association. Furthermore, we did not observe any significant (F = 0.343, *p*-value = 0.570) association between the patients’ age and the duration of the RBD symptoms.

### HEP amplitudes are reduced across wake-sleep stages

**Fig. 3:**
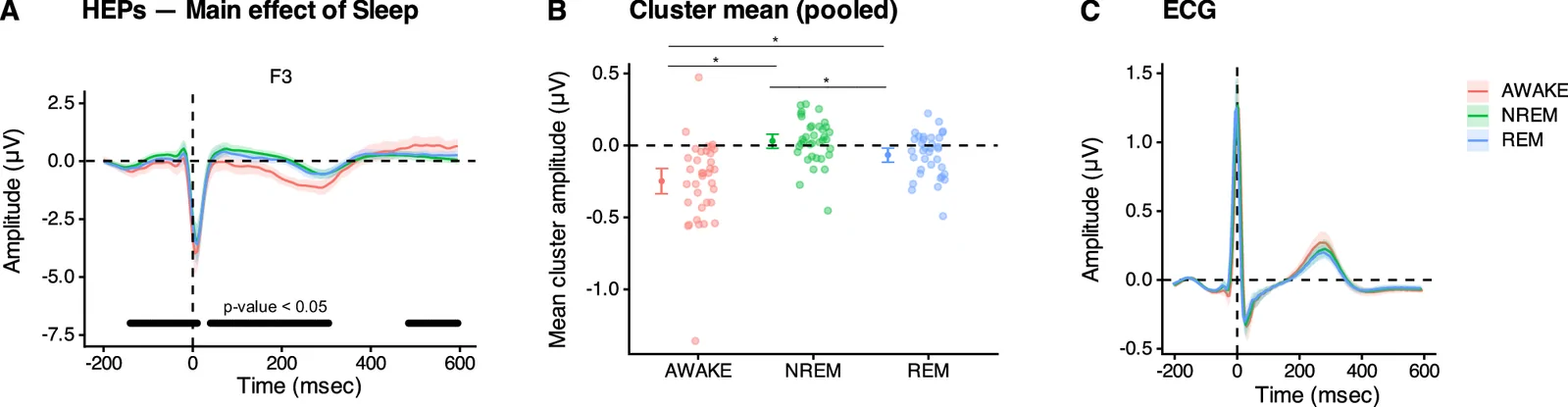
HEP amplitudes modulations across wake-sleep stages. **A** HEPs on electrode F3 (part of the significant cluster), during wakefulness (red), NREM (green), and REM (blue). HEP amplitude is significantly reduced during NREM, and REM sleep in comparison to wakefulness. The horizontal black line represents the period of a significant main effect of sleep (*p*-values < 0.05; FDR-corrected) obtained through linear mixed models. The continuous lines represent the groups’ means, and the shaded areas represent the 95% CI. **B** Mean HEP amplitude over the period showing a significant main effect of sleep. Post-hoc analyses showed a significant difference (*p*-value < 0.05) between wakefulness and sleep stages, and between sleep stages. Each dot represents a subject; the larger dot represents the mean with the 95% CI. **C** ECG amplitude. We did not observe any main effect of wake-sleep *(p*-values > 0.05; FDR-corrected).

Based on previous evidence from healthy young individuals showing that HEP amplitude is reduced during sleep when compared to wakefulness^48^, we examined whether this modulation is also present in our elderly cohort. Our results indicate that HEP amplitude is significantly (*p*-value < 0.05; FDR-corrected; Fig. 3A) modulated by the wake-sleep stages (main effect), over all fronto-central electrodes (F3, F4, C3, and C4; Suppl. Figure 1; Suppl. Table 2). Post-hoc analyses showed a significant difference in HEP amplitude between the three stages (wakefulness, NREM, and REM), all *p*-values < 0.01 (Fig. 3B).

To rule out the possibility that these HEP differences are related to ECG differences, we also assessed whether the ECG signal amplitude is influenced by sleep-wake stages. The findings reveal no statistically significant effect (*p*-value > 0.05; FDR-corrected) (Fig. 3C), indicating that the changes in HEPs observed across sleep-wake stages are not simply due to peripheral cardiac differences.

### Heart rate and inter-beat interval are modulated by the wake-sleep cycle, but not by iRBD

The repeated-measures permutation ANCOVA revealed significant main effects of sleep stage on heart rate (F = 17.21, *p*-value = 0.001; Suppl. Table 3 and Suppl. Table 4) and mean interbeat interval (F = 18.79, *p*-value = 0.001; Suppl. Table 3 and Suppl. Table 4). The normalized frequency-domain indices pLF and pHF showed a stage-dependent trend that did not survive FDR correction (F = 4.17, *p*-value = 0.068; Suppl. Table 3 and Suppl. Table 4). As expected, pHF was numerically highest in NREM (HC: 35.9%; iRBD: 42.9%) and lower in awake (HC: 28.9%; iRBD: 33.7%) and REM (HC: 26.3%; iRBD: 34.9%), consistent with parasympathetic predominance during NREM sleep^49^. Total spectral power, absolute LF and HF power, and time-domain metrics (RMSSD, SDNN, SDSD, pNN50) did not show significant stage modulation. No significant Group differences were observed for any HRV metric after age adjustment. Group main effects and Group X wake-sleep interactions were not significant (Suppl. Table 3 and Suppl. Table 4). Because periods of apnea and hypopnea were removed prior the analyses they cannot account for these results.

## Discussion

iRBD is recognized as one of the earliest manifestations of α-synucleinopathy, often preceding the onset of PD or DLB. While emerging evidence points to potential alterations of interoceptive signals in patients with iRBD, this area remains largely unexplored. In the present study, leveraging whole-night polysomnography recordings, we analyzed brain processing of the heartbeat signal (Fig. 1), reflected by HEP recordings in iRBD patients vs. HC to determine the presence of altered brain-body coupling in iRBD patients. First, we report that during wakefulness, iRBD patients have altered HEP amplitude, but not during NREM and REM sleep stages (Fig. 2). Second, the duration of the RBD symptoms is positively associated with the HEP amplitude alteration observed during wakefulness. Third, the HEP amplitude is decreased during sleep (compared to wakefulness; Fig. 3). Fourth, we observed alterations in cardiac activity (heart rate and inter-beat interval) when comparing wakefulness with sleep but did not observe any modulation in iRBD (Suppl. Table 3, 4). Taken together, our findings show sleep-wake stage-specific alterations in markers for brain-body coupling related to iRBD. Such altered brain–body coupling may reflect underlying neuropathology and highlight HEP as a promising quantitative biomarker for iRBD.

Several pathophysiological mechanisms associated with iRBD may account for the altered HEPs we observed. First, iRBD has been associated with structural and functional alterations in the brainstem and thalamus^7^^,^^40^^,^^49–51^. These two brain regions have been identified as key subcortical hubs receiving afferent neural interoceptive signals coming from the heart^31,32,52^. Therefore, we might interpret the altered HEP amplitude, observed in iRBD patients, as the result of the overlap between the core iRBD pathophysiological and the interoceptive processing pathways. Second, iRBD is also characterized by cortical alterations, including the sensorimotor cortex and frontal and posterior cortical regions^35,37,38^. Some of these regions, especially the frontal, have been associated with HEP^32,46^, and their alterations might explain the altered HEP amplitude we observed in iRBD patients. Third, according to the recent body-first hypothesis, α-synuclein pathology may originate in peripheral organs and propagate to subcortical and cortical regions via the vagus nerve^33,34,53–55^. The vagus nerve conveys interoceptive signals, such as those coming from the heart, from the organs to the brainstem; therefore, vagus nerve alterations due to the accumulation of α-synuclein might underlie the present HEP alterations. Fourth, the HEP alterations observed in our data might not only come from alterations in the brainstem and interoceptive pathways, but they might also arise due to the cardiac sympathetic denervation that has been observed in virtually every patient with iRBD^13,56,57^. Contributions by several of the indicated systems likely contribute to producing the observed HEP changes in iRBD. Moreover, we note that our results showing HEP alterations during wakefulness are not related to changes in the ECG signal nor ECG features (such as HRV). This is important and shows that the HEP changes are rather related to alterations in neural interoceptive pathways. Our results were not influenced by periods of apnea, hypoapnea nor microarousal, and none of the patient reported symptoms of orthostatic hypotension, which might have affected the EEG signal or the HEP.

In line with previous work showing that HEPs are more prominent over fronto-central regions in healthy participants^32,47^, we observed that HEP amplitude is modulated over frontal regions (as a function of Group, but also wake-sleep stage). Furthermore, our results show a significant difference between iRBD and controls in the left frontal region. Growing evidence indicates that subcortical and cortical neural degeneration in iRBD is predominantly on the left side^58–60^. Together with evidence that cortical cardiac autonomic control may involve the left anterior insula^61^, this might (at least partially) explain our results. However, additional studies combining MRI and EEG are necessary to corroborate this hypothesis. Our results also show a modulation of the HEP amplitude based on the wake-sleep stage, with a decreased amplitude during sleep compared to wakefulness. Sleep-related HEP amplitude attenuation is thought to reflect reduced cortical responsiveness to visceral inputs, driven by state-dependent changes in thalamocortical dynamics and cortical excitability^48,62^.

Our results show that the HEP amplitude changes between the two groups were observed during wakefulness but not during sleep. These results align with previous findings and extend to clinical populations the evidence that HEP amplitude is not modulated by nightmares. That is, previous results showed no HEP amplitude changes when comparing individuals with nightmares with those without^63^ (but see ref. ^64^). This may be explained by an overall reduction in HEP amplitude during these stages (compared to wakefulness), which could mask between-group differences due to the limited signal strength during sleep. Furthermore, the HEP amplitude modulation based on the wake-sleep stage occurred pre and post ECG R-peak. The difference in HEP amplitude occurring before the R-peak has been associated with anticipatory mechanisms^65^, and is compatible with the view that the cardiac cycle, and therefore HEPs, is a continuous sequence of events without a distinct beginning or end.

Our analysis of cardiac activity across wakefulness and sleep stages revealed a pronounced effect on the heart rate and inter-beat interval of vigilance state, independent from the group (iRBD and controls). This pattern is consistent with a large body of evidence showing that heart rate and inter-beat interval are significantly modulated by vigilance state^66^. Furthermore, we did not observe differences in HRV between iRBD patients and controls, nor a significant interaction between Group and wake-sleep. These findings are consistent with previous work failing to show HRV alterations in iRBD patients^19,20^. Yet, some studies reported reduced HRV or altered sympatho-vagal balance in iRBD^18^. Taken together, our HRV results argue against a pronounced, globally altered autonomic state in iRBD and, therefore, do not account for the HEPs alterations observed in iRBD.

In conclusion, our results show that altered brain-heart interactions, indexed by HEPs, are a new quantitative marker for iRBD and the duration of the RBD symptoms. These results shed light on the possible use of HEP as a quick, accessible, and cost-effective marker for RBD. HEP alteration might already be present at the moment of early vagal symptoms, even in the absence of clinical RBD, possibly being also a prodromal marker.

Our findings should be considered in the context of the following limitations. First, the results are promising and should be replicated in a larger sample of patients. Second, although the present analysis corrected for multiple comparisons across each time point within the epoch, the spatial extent of the analysis was not pre-specified. Future studies should adopt a confirmatory approach by defining a priori spatio-temporal regions of interest based on the present findings and control for multiple comparisons across both time and channels simultaneously. Third, although our results show effects with a reduced number of electrodes (*n* = 6), high-density EEG recordings would allow a more precise localisation of the HEP, as well as the possibility to further remove possible artefacts which might be present in the signals (e.g., pulse artifact). Fourth, although we did not observe a difference in the ECG signal, future studies should investigate whether the groups have no difference in cardiac functions through specific tests, such as cardiac scintigraphy. This would ensure that the difference in HEP amplitude is related to a difference in neural response and not arising from a difference in cardiac activity. Fifth, although we report several medications, future studies should also investigate and include medications affecting autonomic function. Sixth, none of the participants included here reported symptoms of orthostatic hypotension, yet quantitative measures should be performed systematically in all patients in future studies. Seventh, longitudinal data are needed to corroborate the predictive role of HEP for the conversion from iRBD to PD or DLB.

## Methods

### Participants

Thirty-six datasets were included in the present analysis, comprising recordings obtained at the outpatient clinic of the Sleep–Wake–Epilepsy Center (SWEC) at Inselspital, University Hospital Bern, Switzerland. Thirteen datasets were from patients diagnosed with isolated REM sleep behavior disorder (iRBD), and twenty-three were from healthy control participants. All iRBD diagnoses were established by a neurologist specialized in sleep medicine. Exclusion criteria included a history of major psychiatric disorders, cerebrovascular disease, or any condition known to affect mental status. None of the participants had used antipsychotic medication within 5 years before the polysomnographic recording. No significant difference was observed regarding the use of antihypertensive medications (iRBD: 6/13 vs HC: 10/23, *p*-value = 1.000; Fisher’s exact test), antidepressant (iRBD: 4/13 vs HC: 7/23, *p*-value = 1.000; Fisher’s exact test), nor for dopaminergic medications (iRBD: 0/13 vs HC: 1/23, *p*-value = 1.000; Fisher’s exact test).

### Ethics declaration

The study protocol was approved by the local cantonal ethics committees (Bern BASEC #2022-01379 and 2021-00619; Geneva: #2019-02275), following the Swiss Federal Act on Research involving Human Beings (Human Research Act, HRA), the Ordinance on Human Research with the Exception of Clinical Trials (Human Research Ordinance, HRO, Chapter 2), and the Swiss Federal Act on Data Protection (Data Protection Act, FADP). Written informed consent was obtained from all participants.

### Polysomnography recordings and visual analysis

Overnight video-polysomnography (PSG) recordings were acquired using the *REMLogic* system (Embla Systems) and included bilateral frontal, central, temporal, and occipital electroencephalography (EEG) electrodes referenced online to Cz, bilateral electrooculography (EOG), and submental as well as anterior tibialis electromyography (EMG). PSG recordings were obtained between 2012 and 2021 and scored according to the criteria of the American Academy of Sleep Medicine (AASM 3). The diagnosis of iRBD was established according to the *International Classification of Sleep Disorders*, Third Edition (ICSD-III). One individual in the control group had ≤ 1% REM sleep. For the iRBD patients the lowest REM sleep was 9.5%. REM sleep behaviors were classified as minor, major, complex, or scenic, and vocalizations were documented.

### Automated sleep staging

To improve reproducibility and overcome inter-rater variability^67^, PSGs were automatically analysed to identify sleep stages using ‘Yet Another Spindle Algorithm’ (YASA) algorithm^68^. Sleep recordings were divided into 30-second epochs, and the previously validated algorithm with integral artifact rejection was used to automatically classify sleep stages based on scalp EEG (C4). The 30-s EEG epoch was classified as either wakefulness, N1 sleep, N2 sleep, N3 sleep, or REM sleep. YASA algorithm provides the probability (i.e., likelihood) for each epoch of each stage, informing about the confidence level of the algorithm in identifying a sleep stage. The assessment of HEP amplitude differences across groups and sleep stages was conducted based on the YASA sleep staging algorithm. Pairwise agreement between YASA automated staging and manual scoring was computed at the 30-second epoch level. For each epoch, the modal stage value was extracted from the sample-by-sample staging vectors and compared between the two systems. Agreement was expressed as the percentage of epochs assigned the same stage label by both systems. We observed 73.2 ± 11.4% (mean ± SD) of agreement between the manual sleep stage scoring and YASA automated sleep stage scoring.

### R-peak detection and Heart Rate Variability

ECG was recorded concurrently with polysomnography and down sampled to 200 Hz. R-peaks were detected using a maximal overlap discrete wavelet transform (MODWT; sym4 wavelet, decomposition levels 3–5) to enhance QRS complexes, followed by peak detection in sliding 60 s windows with per-window normalization and an adaptive prominence threshold (mean + 3 SD, bounded between 0.05 and 0.6 on the normalized scale). R-R intervals outside physiological bounds (0.4–1.5 s, corresponding to 40–150 bpm) were discarded. Remaining intervals were further cleaned using a relative threshold: beats deviating by more than 20% from the running 7-beat median, or showing successive differences greater than 25% of the local median, were flagged and replaced by linear interpolation.

Heart rate variability was computed separately for each wake-sleep stage following Task Force (1996) recommendations. Time-domain metrics included the mean inter-beat interval (IBI), mean heart rate (HR), the standard deviation of NN intervals (SDNN), the root mean square of successive differences (RMSSD), the standard deviation of successive differences (SDSD), and the percentage of successive NN differences exceeding 50 ms (pNN50). For frequency-domain analysis, the tachogram was interpolated at 4 Hz using cubic-spline interpolation, linearly detrended, and submitted to Welch’s periodogram estimation (Hanning window, 50% overlap). Absolute power was integrated over the very-low (VLF; 0.003–0.04 Hz), low (LF; 0.04–0.15 Hz), and high-frequency bands (HF; 0.15–0.40 Hz) and expressed in ms². Normalized indices were computed as pLF = 100 × LF / (LF + HF) and pHF = 100 × HF / (LF + HF), together with the LF/HF ratio and total spectral power (TP = VLF + LF + HF). Heart rate variability (HRV) was calculated on the continuous data, separately for each sleep stage. Analysis of the cardiac activity was done on the continuous signal after removal of periods of apnea/hypopnea.

### Preprocessing

PSG recordings underwent a thorough artifact removal procedure; data were recorded with a sampling frequency of 200 Hz. First, the EEG continuous data were bandpass filtered between 0.5 Hz and 40 Hz (Butterworth of 5^th^ order) and then referenced to the two Mastoids. We epoched the continuous polysomnography data using the ECG R-peak as a time-locking event. Epochs included a window of −200 ms and +600 ms centred on the R-peak. Epochs in which the EEG signal amplitudes were higher than ± 75 μV were rejected. Finally, epochs were visually inspected. Given the view that the cardiac cycle, and therefore HEPs, is a continuous sequence of events without a distinct beginning or end, we did not apply any baseline correction. This avoids the post-R-peak HEPs modulation results from a baseline correction.

### Apnea and hypopnea

Periods of apnea and hypopnea were identified from the nasal cannula pressure using an automated detection algorithm implemented in MATLAB, following AASM scoring criteria. The airflow signal was bandpass filtered between 0.05 and 2 Hz using a third-order Butterworth filter to isolate the respiratory band and remove DC drift. A rolling root mean square (RMS) envelope was computed over a 2-second sliding window to obtain a smooth, non-negative amplitude estimate at each time point. To account for inter-individual differences in signal amplitude and within-night variation in breathing depth, the envelope was normalised against a 120-second rolling median baseline computed exclusively from sleep periods, rendering the detection scale-free across subjects and recording conditions. Apnea was defined as a reduction in normalised airflow amplitude below 10% of baseline ( ≥ 90% reduction) sustained for at least 10 seconds; hypopnea was defined as a reduction below 70% of baseline ( ≥ 30% reduction) sustained for at least 10 seconds. Detected event periods, plus a 1.5-second post-event buffer to capture associated arousal and movement artefacts, were subsequently removed from all EEG channels.

### Statistical analyses for demographic and clinical variables

Statistical differences between iRBD and HC regarding demographic variables were assessed using the unpaired t-test and Fisher’s exact test. RBD duration was calculated as the number of years between the PSG recording date and the onset of RBD symptoms reported by the patients. Because the onset of symptoms is not always precise (due to the subjective report from the patient), we set the beginning date to July 1 of the year indicated by each patient. For two patients, the duration was negative because the arbitrary onset date of July 1 occurred after the date of the PSG recordings. For these two patients, the duration was therefore set to zero.

### Statistical analyses for Heartbeat evoked potentials (HEPs)

We computed the HEPs for each EEG channel (F3, F4, C3, C4, O1, O2) and sleep stage (awake, NREM, and REM). To assess HEP amplitude alterations in iRBD patients, we computed linear mixed models for each channel, time point (i.e., every 5 ms), and sleep stage independently. We used amplitude as the dependent variable and group (iRBD vs. HC) and sleep stage (awake, NREM, and REM) as fixed effects and added an interaction between the two terms. Age was included as a covariate of no interest, and a random intercept was added for each individual to account for inter-individual variability. We also added a random slope for the wake-sleep stage. This statistical model was applied to the window −200ms to +600 ms, locked to the ECG R-peak. The same analyses were computed for the ECG channel. Correction for multiple comparisons of the epoch length (-200ms to 600 ms) was obtained using the False Discovery Rate (FDR)^69^. FDR was applied to each channel independently, and for each effect (interaction and main effects). Following identification of significant Group × wake-sleep clusters in the timepoint-by-timepoint analysis, per-subject cluster means were computed by averaging amplitudes across all (channel × time) coordinates within significant clusters, separately per sleep stage. Post-hoc analyses were performed on the mean of the significant periods (providing one values per subject), using a linear regression with HEP amplitude dependent variable, Terms (either Group or wake-sleep stage) as independent variable, and Age as a covariate of no interest

During wakefulness we recorded 2402 ± 990 (mean ± SD) HEPs for the iRBD and 2533 ± 1763 for HC (p-value = 0.777; t(1,34) = 0.285). During the NREM sleep stage, we recorded 12,858 ± 2523 epochs for the iRBD and 13,145 ± 4635 for HC (*p*-value = 0.812, t(1,34) = 0.24). In the REM phase, we observed 2768 ± 1726 epochs and 3677 ± 1814 for HC (*p*-value = 0.163, t(1,24) = 1.442).

### Association RBD symptoms duration and HEP amplitude

To investigated whether the duration of the RBD symptoms is associated with the HEP amplitude alterations observed during wakefulness in iRBD patients, we conducted robust regression (which presents the advantage of being less affected by outliers^70^) between the duration of the RBD symptoms (from the onset of the symptoms till the day of the PSG; as dependent variable) and the HEP amplitude (mean of the 305 ms–445 ms significant cluster for electrode F3).

### Statistical analyses for Heart rate variability (HRV)

Because HRV metrics were moderately skewed, group comparisons were conducted using permutation-based (5000 permutations) repeated-measures ANCOVA, implemented in the permuco R package^71^, which avoids distributional assumptions while preserving the within-subject dependence structure. The primary model tested the fixed effects of Group (iRBD vs HC), wake-sleep stage (awake, NREM, REM), their interaction, and age as a covariate. *P*-values were adjusted for multiple comparisons using the Benjamini-Hochberg false discovery rate (FDR) across the HRV metrics within each effect term.

## Supplementary information

Supplementary_Bernasconi_NPJ_PD_round2_June_2026.

## Data Availability

Raw data sharing will require a formal data use agreement. Requests will be evaluated based on institutional and departmental policies to determine whether the data requested is subject to intellectual property or patient privacy obligations. Data to reproduce the main figures are available via https://gitlab.epfl.ch/fbernasc/irbd_hep_npj.

## Code availability

Codes are available https://gitlab.epfl.ch/fbernasc/irbd_hep_npj.
