# Supplementary material for: Altered heart-brain coupling in awake patients with isolated REM sleep behaviour disorder
Source: NPJ Parkinsons Dis. 2026 Jul 23;12:282. doi: 10.1038/s41531-026-01485-7 (PMC13759489; doi:10.1038/s41531-026-01485-7)
Supplement: Supplementary file 1 [file 41531_2026_1485_MOESM1_ESM.pdf]

Supplementary information

Title

Altered heart-brain coupling in awake patients with isolated REM sleep behaviour disorder

Authors

Fosco Bernasconi[1], Julia van der Meer[2], Zehra Merchant[1], Jevita Potheegadoo[1], Marzia De Lucia[4,5], Claudio Bassetti[2], Carolin Schaefer[2,6,7], Olaf Blanke[1,3,7]

Affiliations

1. Laboratory of Cognitive Neuroscience, Center for Neuroprosthetics & Brain Mind Institute, Ecole Polytechnique Fédérale de Lausanne (EPFL), Geneva, Switzerland
2. Sleep-Wake Epilepsy Center, Department of Neurology, Inselspital, University Hospital of Bern, University of Bern, Switzerland
3. Department of Neurology, Geneva University Hospitals, Geneva, Switzerland
4. Brain-Body and Consciousness Laboratory, Department of Clinical Neuroscience, Lausanne University Hospital, University of Lausanne, Lausanne, Switzerland
5. Center for Biomedical Imaging, Lausanne, Switzerland
6. Clinic for Sleep Medicine, KSM Luzern, Lucerne, Switzerland
7. These authors have contributed equally

Corresponding authors:

Dr. Fosco Bernasconi
École Polytechnique Fédérale de Lausanne
Neuro-X Institute
Campus Biotech H4.02
Ch. des Mines 9
1202 Geneva
fosco.bernasconi@gmail.com

Prof. Olaf Blanke
École Polytechnique Fédérale de Lausanne
Neuro-X Institute
Campus Biotech H4.02
Ch. des Mines 9
1202 Geneva
Olaf.blanke@epfl.ch

HEPs are altered in iRBD during wakefulness, but not during NREM and REM sleep

To corroborate the relevance of the left lateralization for the interaction Group X wake-sleep stage (i.e., effect on F3 for the difference in HEP amplitude between iRBD and controls during wakefulness), we computed additional analyses by investigating the HEP amplitude for a left cluster (F3, C3) and for the right cluster (F4, C4). Our results showed a significant interaction Group X Wake-sleep stage, over the period 320-450ms after the R-peak for the left cluster. No significant interaction was observed for the right cluster.

Periods of apnea and hypopnea

Metric	iRBD	HC	p-value
NREM Apnea Events	7.231 ± 20.733	0.435 ± 1.879	0.261
NREM Hypopnea Events	7.769 ± 17.059	12.130 ± 35.173	0.788
REM Apnea Events	0.538 ± 1.127	0.261 ± 0.915	0.457
REM Hypopnea Events	3.231 ± 5.615	3.217 ± 7.026	0.995
AHI Total	4.216 ± 7.896	3.564 ± 10.245	0.833

Table 1. Number of events of apnea and hypopnea. The number of apnea and hypopnea events is not significantly different between the two groups. The Apnea-Hypopnea Index (AHI) is also not different between the two groups. P-values are obtained from a Welch t-test.

HEP – Main effect of wake-sleep stages

Channel	Cluster 1	Cluster 2	Cluster 3	Cluster 4	Cluster 5
C3	-95—85 ms	-55—35 ms	-25–10 ms	40–310 ms	495–595 ms
C4	-50–10 ms	40–290 ms	485–595 ms		
F3	-140–10 ms	40–305 ms	485–595 ms		
F4	-175—135 ms	-125–10 ms	35–295 ms	460–595 ms	

Table 2. Significant cluster showing a modulation of HEPs amplitude as a function of sleep. The table indicates the periods during which a main effect of sleep was observed, for each electrode separately.

HEP amplitudes are reduced across wake-sleep stages

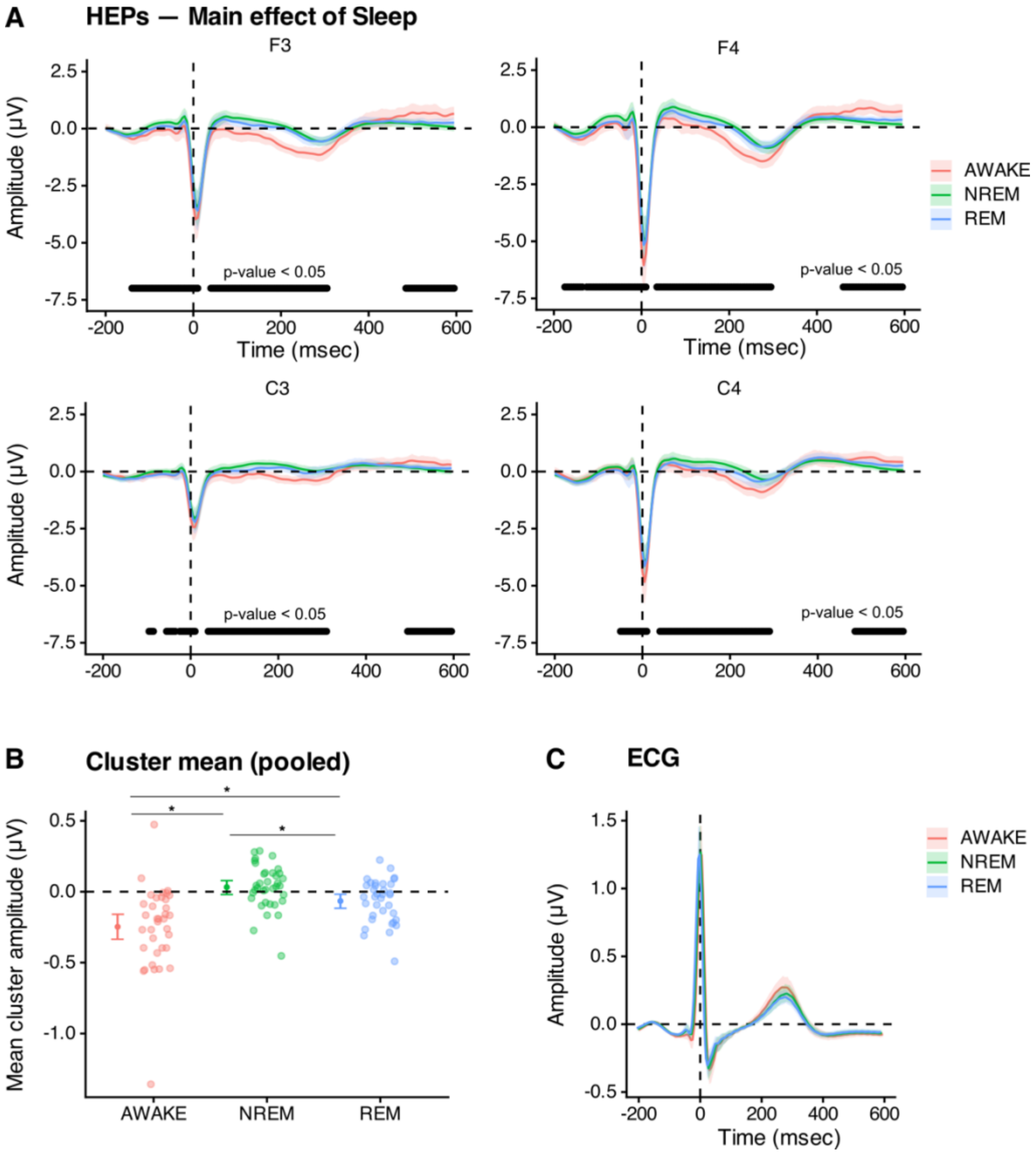

Figure 1. HEP modulation across sleep-wake stages. A. HEPs on electrodes F3, F4, C3, and C4 during wakefulness (red), NREM (green), and REM (blue). HEP amplitude is significantly reduced during NREM and REM sleep stages compared with wakefulness. The horizontal black line indicates the period with a significant main effect of sleep (p-values < 0.05, FDR-corrected) obtained through linear mixed models. Continuous lines represent the group means, and shaded areas represent the 95% CI. **B.** Mean HEP amplitude over the period showing a significant main effect of sleep. Post hoc analyses showed a significant difference (p-value < 0.05) between wakefulness and sleep stages, and between sleep stages. Each dot represents a subject; the larger dot represents the mean with the 95% CI**. C.** ECG amplitude. We did not observe any significant difference (p-values > 0.05; FDR-corrected).

Heart rate variability (HRV)

HRV metric	AWAKE		NREM		REM	
	iRBD	HC	iRBD	HC	iRBD	HC
Heart rate (bpm)	62.1 (56.7–66.7)	61.3 (56.4–70.6)	61.4 (51.9–64.1)	57.9 (55.7–68.5)	60.5 (51.6–63.5)	59.4 (57.4–67.9)
Mean IBI (s)	0.97 (0.90–1.06)	0.98 (0.85–1.06)	0.98 (0.94–1.16)	1.04 (0.88–1.08)	0.99 (0.95–1.16)	1.01 (0.88–1.05)
RMSSD (ms)	28.70 (17.99–35.70)	28.65 (18.67–39.06)	29.32 (22.71–43.79)	23.40 (17.38–39.05)	32.44 (20.01–46.84)	25.94 (17.19–32.58)
SDNN (ms)	58.45 (41.34–72.16)	50.04 (41.61–89.90)	67.50 (42.85–76.15)	53.39 (43.90–63.41)	59.41 (45.14–69.50)	57.22 (52.75–84.67)
SDSD (ms)	28.70 (18.00–35.70)	28.66 (18.68–39.07)	29.32 (22.71–43.79)	23.40 (17.38–39.05)	32.44 (20.01–46.82)	25.94 (17.19–32.58)
pNN50 (%)	2.7 (0.9–9.1)	5.5 (1.2–11.3)	3.5 (0.8–14.2)	4.1 (0.8–12.1)	2.0 (1.0–18.4)	3.6 (1.2–9.0)
LF power (ms²)	424 (201–1180)	395 (263–934)	509 (243–880)	272 (158–676)	484 (260–947)	491 (196–818)
HF power (ms²)	170 (106–375)	247 (96–448)	117 (97–501)	180 (85–328)	171 (98–507)	185 (71–305)
Total power (ms²)	1710 (1055–3411)	1721 (1101–3161)	2670 (938–4119)	1366 (819–1985)	2229 (1146–3469)	2119 (1044–3373)
LF proportion (%)	66.3 (60.9–69.7)	71.1 (63.8–77.4)	57.1 (54.6–75.3)	64.1 (50.4–71.7)	65.1 (54.8–79.4)	73.7 (59.1–81.9)
HF proportion (%)	33.7 (30.3–39.1)	28.9 (22.6–36.2)	42.9 (24.7–45.4)	35.9 (28.3–49.6)	34.9 (20.6–45.2)	26.3 (18.1–40.9)
LF/HF ratio	1.97 (1.56–2.30)	2.47 (1.76–3.43)	1.33 (1.20–3.05)	1.79 (1.02–2.53)	1.87 (1.21–3.86)	2.80 (1.45–4.51)

Table 3. HRV metrics by group and sleep stage. IBI = inter-beat interval; RMSSD = root mean square of successive differences; SDNN = SD of NN intervals; SDSD = SD of successive differences; pNN50 = % successive NN differences >50 ms; LF = 0.04–0.15 Hz power; HF = 0.15–0.40 Hz power; TP = total power (0.003–0.40 Hz); pLF, pHF = LF and HF as % of (LF+HF); LF/HF = ratio. Values are median (interquartile range, Q1–Q3).

HRV metric	Patient (iRBD vs HC)			Sleep (AWAKE/NREM/REM)			Patient × Sleep		
	F	p	pFDR	F	p	pFDR	F	p	pFDR
Heart rate (bpm)	0.00	.961	.961	17.21	<.001	**.001**	0.95	.388	.753
Mean IBI (s)	0.01	.924	.961	18.79	<.001	**.001**	1.84	.173	.691
RMSSD (ms)	0.09	.767	.961	0.08	.920	.999	2.53	.093	.560
SDNN (ms)	0.35	.567	.961	0.00	.997	.999	1.25	.300	.753
SDSD (ms)	0.09	.766	.961	0.08	.923	.999	2.53	.083	.560
pNN50 (%)	0.29	.589	.961	0.00	.999	.999	0.37	.693	.758
LF power (ms²)	0.00	.956	.961	1.26	.294	.588	1.09	.349	.753
HF power (ms²)	0.18	.688	.961	0.59	.571	.895	0.85	.439	.753
Total power (ms²)	0.50	.541	.961	0.72	.596	.895	0.63	.695	.758
LF proportion (%)	0.35	.565	.961	4.17	.023	.068	0.43	.638	.758
HF proportion (%)	0.35	.558	.961	4.17	.022	.068	0.43	.651	.758
LF/HF ratio	0.10	.762	.961	2.01	.128	.307	0.14	.896	.896

Table 4. Permutation repeated-measures ANCOVA for HRV metrics. P-values from permutation tests. pFDR = FDR-adjusted across HRV metrics.
